# Impact of MMR status on preoperative CT-based lymph node overstaging in right-sided colon cancer: a retrospective analysis

**DOI:** 10.1186/s40644-026-00992-3

**Published:** 2026-02-05

**Authors:** Zexian Chen, Yuting Zhang, Hao Chen, Yanyun Lin, Hui He, Bin Zhang, Yongle Chen

**Affiliations:** 1 Department of General Surgery (Colorectal Surgery), The Sixth Affiliated Hospital, Sun Yat-sen University, Guangzhou, Guangdong 510655 People’s Republic of China; 2 Guangdong Provincial Key Laboratory of Colorectal and Pelvic Floor Diseases, The Sixth Affiliated Hospital, Sun Yat-sen University, Guangzhou, 510655 People’s Republic of China; 3 Biomedical Innovation Center, The Sixth Affiliated Hospital, Sun Yat-sen University, The State Key Laboratory of Oncology in South China, Guangzhou, 510655 People’s Republic of China; 4 Department of Oncology, The Sixth Affiliated Hospital, Sun Yat-sen University, Guangzhou, 510655 People’s Republic of China; 5 The Sixth Affiliated Hospital, Sun Yat-sen University, 26 Yuancun Erheng Road, Guangzhou, Guangdong 510655 China

**Keywords:** Mismatch repair, Lymph node staging, Colorectal cancer, Overstaged, Right-sided colon cancer

## Abstract

**Background:**

Neoadjuvant treatment for colon cancer has recently gained more and more attention. Given the imprecision of radiologic staging, low-risk patients may be exposed to the toxicities and inconvenience of chemotherapy when surgery alone might have been considered sufficient. Overstaged lymph nodes via computed tomography (CT) may lead to unnecessary neoadjuvant treatment. However, the clinical features and prognostic factors of preoperative CT-based lymph node overstaging in right-sided colon cancer, particularly the impact of mismatch repair (MMR) status, are still not well understood.

**Methods:**

This single-center retrospective cohort study enrolled 1687 patients with nonmetastatic right-sided colon cancer who underwent curative resection for primary colorectal lesions between 2013 and 2020 at the Sixth Affiliated Hospital of Sun Yat-sen University. Of these, 635 patients were assigned to the overstaged group, whereas 235 patients were assigned to the understaged group. We investigated the preoperative clinical features of overstaged groups using univariate logistic regression analysis and nomogram logistics analysis. The multivariate Cox regression analysis was used to explore the prognostic factors of the overstaged group.

**Results:**

The deficient mismatch repair (dMMR) patients with right-sided colon cancer were more likely to be overstaged before surgery (odds ratio [OR], 1.551; 95% confidence interval [CI],1.210–1.989; *P* = 0.001). Overstaged patients had the best disease-free survival (DFS) (*P* = 0.007), while understaged patients had the worst overall survival (OS) (*P* = 0.001) and DFS (*P* < 0.001). Moreover, dMMR status was a protective factor for DFS in the overstaged group (HR = 0.535, 95% CI: 0.307–0.932; *P* = 0.027). Furthermore, carcinoembryonic antigen (CEA) level > 5 ng/mL, perineural invasion, and adjuvant chemotherapy were independent prognostic factors in the overstaged group.

**Conclusion:**

Consequently, the dMMR patients with right-sided colon cancer are more likely to be overestimated via preoperative CT scans. Patients with dMMR had a better prognosis in the overstaged group. The tendency towards nodal overstaging in dMMR right-sided colon cancer indicated there should be a more optimal assessment of CT-based lymph node staging combined with the MMR status.

**Supplementary Information:**

The online version contains supplementary material available at 10.1186/s40644-026-00992-3.

## Introduction

Colorectal cancer (CRC) is the leading cause of cancer-related death in men and the second leading cause of death in women younger than 50 years in the USA [1]. Moreover, CRC ranks third in new cases and deaths among all malignant tumors, with its incidence and mortality rates rising annually in China [2]. Lymph node metastasis is an important basis for evaluating CRC prognosis. Accurate assessment of the lymph node metastasis status is crucial for the prognosis and outcome of patients with colorectal cancer.

Computed tomography (CT) is the preferred method for the primary staging of colon cancer, especially for ruling out metastatic disease. However, CT is unreliable for locoregional staging (T stage and N stage) of colon cancer, and its overall accuracy in determining lymph node metastasis is 57–67% [3–5]. Preoperative staging with magnetic resonance imaging (MRI) for rectal cancer has led to the standard use of neoadjuvant radiochemotherapy. In contrast, FOxTROT and PRODIGE 22 trials about neoadjuvant chemotherapy for colon cancer have categorized low-risk tumors as high risk on the basis of CT scans [6–8]. The accuracy of lymph node staging in colon cancer is lower, limiting the use of neoadjuvant recommendations.

Benign mesenteric lymph node enlargement confirmed by postoperative histopathological examination is commonly observed in clinical practice. This condition can be a useful positive factor in predicting recurrence and long-term survival in patients with colorectal cancer [9]. Studies have shown that right-sided colon cancer patients often have the highest lymph node yield [10, 11]. CT scans of right-sided colon cancer patients revealed that partially benign mesenteric lymph node enlargement was malignant [5]. However, the clinical features and prognostic factors of right-sided colon cancer with overestimated lymph node staging remain unknown.

Microsatellite stability is sustained through a process called mismatch repair (MMR), which corrects the DNA mismatches generated during DNA replication. However, deficient mismatch repair (dMMR) causes microsatellite instability owing to the accumulation of errors in the microsatellite sequences [12, 13]. The CT imaging features of primary colon cancer differ between dMMR and proficient mismatch repair (pMMR) tumors, suggesting that the assessment of CT-based colon cancer staging should consider the MMR status, particularly regarding lymph node evaluation [14]. Therefore, this study aimed to determine the impact of MMR status on the overestimation of lymph node staged in right-sided colon cancer patients.

## Materials and methods

### Study design and patients

This retrospective cohort study included patients with histologically confirmed stage I‌-III‌ right-sided colon cancer who underwent curative surgery at The Sixth Affiliated Hospital of Sun Yat-sen University between 2013 and 2020. Patients with missing MMR status, CT N-stage, adenocarcinoma in situ or high-grade intraepithelial neoplasia, neoadjuvant chemotherapy, or insufficient information were excluded. The patients were divided into consistent, overstaged, and understaged groups on the basis of their preoperative CT N stage. The overstaged group was defined as having a higher preoperative CT N-stage than postoperative pathological N-stage. The understaged group was defined as having a lower preoperative CT N-stage than postoperative pathological N-stage. The consistent group was defined as those for whom the preoperative CT N-stage was consistent with the postoperative pathological N-stage.

This retrospective cohort study was approved by the Ethics Committee of the Sixth Affiliated Hospital of Sun Yat-sen University (No. E2023148). All methods were performed in accordance with the relevant guidelines and regulations.

### CT scan image and MMR status analysis

All patients received abdominopelvic contrast-enhanced CT, using OPTIMA CT660 (GE Medical Systems, Milwaukee, WI, United States) or AQUILION ONE (TOSHIBA Medical Systems, Japan) scanner. The acquisition parameters were as follows: tube voltage of 120 kV; tube current of 150–550 mA; matrix of 512 × 512; a gantry rotation time of 0.5s; pitch of 0.97 to 0.99. Nonionic contrast (Ultravist 370, Bayer Health Care (Whippany, NJ, USA)) at the dose of 1.2–1.5 mL/kg weight were injected at a speed of 2.5–3 mL/s with a high-pressure pump syringe. Arterial phase was obtained after 25–30 s of delay after intravenous injection of contrast material, and portal venous phase was performed after 55–70 s of delay. All CT image data were derived from the Picture Archiving and Communication System (PACS). Imaging feature analysis of the primary colon tumour and regional lymph nodes was read retrospectively for the study by two radiologists (senior attending radiologists, with 5 and 8 years of experience respectively) who were blinded to all clinical and histological information (such as MMR status), except the location of the tumour. Lymph nodes with a short diameter > 5 mm, internal heterogeneity and an irregular outer border were considered positive. CT scans were contrast-enhanced in all cases, and no dedicated colon protocols were used. If there was a disagreement between the two radiologists, discussion was performed to reach a consensus.

MMR status was determined using immunohistochemistry (IHC) for protein MLH1, PMS2, MSH2, and MSH6 according to standard protocols. Status of dMMR was defined as the absence of one of these four proteins. Cases with complete nuclear loss of MMR expression in invasive tumor cells but with retained expression in inflammatory cells and/or adjacent normal tissue as positive controls were considered MMR deficiency. The analysis of MMR status was also performed by two pathologists. Just like the CT scans analysis, if there was a disagreement between the two pathologists, discussion was performed to reach a consensus. For equivocal cases such as cases with weakly positive results, detection of MSI status by polymerase chain reaction (PCR) method would be performed.

### Follow-up

After curative surgery, follow-up studies were performed every 3 months for 3 years, and then every 6 months for next 2 years, and annually thereafter, as recommended by Chinese Society of Clinical Oncology (CSCO) guidelines. The follow-up included medical history, physical examination, routine blood tests, comprehensive biochemical examination, thoracic-abdominal-pelvic computed tomography, and colonoscopy. At the same time, we followed the patient’s condition by telephone every six months.

### Statistical analysis

The data for this retrospective analysis were finalized in September 2024. Overall survival (OS) was defined as the time from surgery to death from any cause. Disease-free survival (DFS) was defined as the time interval between surgery and the date of imaging/endoscopic testing, revealing the presence of recurrence or death due to any cause. Recurrence was defined on the basis of pathological, radiological, and clinical examinations. Local recurrence was defined as tumor recurrence in the local area or nearby lymphatic flow area of the surgical operation and adjacent organs, whereas tumors at nonregional sites, such as the liver or lung, were considered distal recurrences. All the data were analyzed via univariate and multivariate logistic regression using SPSS 26.0 statistical software (version 26.0; IBM Corp., Armonk, NY, USA). We analyzed the characteristics of the overstaged group using Logistic Regression and the prognosis using Cox Regression. Differences were considered statistically significant at *p* < 0.05.

## Results

### Patient characteristics and CT accuracy of cN category

A total of 1687 patients with stage I-III right-sided colon cancer, with a median age of 60 years (45.4% women), were included in the analysis after excluding 297 patients. Clinical CT-based cN categories were compared with the final pathology, and N category accuracy was measured as specificity. The specificity of the N stage in patients with right-sided colon cancer was 51.8%, whereas that in dMMR patients was 44.0% (Supplementary Table 1). Consistent with previous research, the N stage was overestimated in half of the patients [15]. In particular, the N stage in patients with dMMR often tended to be overestimated.

According to the clinical CT-based cN categories and final pathology, 635 patients were included in the overstaged group according to preoperative CT staging, whereas 235 patients were included in the understaged group (Supplementary Fig. 1).

T stage, tumor differentiation, tumor histology, perineural invasion, vascular invasion, MMR status, and adjuvant chemotherapy were significantly different among the three groups (Table 1). Furthermore, there was a significant difference in the number of dissected and positive lymph nodes.

**Table 1:** Basic characteristics of patients with stage I-III right-sided colon cancer

	Consistent Group	Overstaged Group	Understaged Group	Total	*P* value
	(cN = pN)	(cN > pN)	(cN < pN)		
	(*N* = 817)	(*N* = 635)	(*N* = 235)	(*N* = 1687)	
**Gender**,** n(%)**					0.851
Female	370(45.3)	292(46.0)	103(43.8)	765(45.3)	
Male	447(54.7)	343(54.0)	132(56.2)	922(54.7)	
**Age**,** n(%)**					0.711
≥50	314(38.4)	240(37.8)	96(40.9)	650(38.5)	
<50	503(61.6)	395(62.2)	139(59.1)	1037(61.5)	
**BMI**,** n(%)**					0.997
<24	576(70.5)	448(70.6)	165(70.2)	1189(70.5)	
≥24	241(29.5)	187(29.4)	70(29.8)	498(29.5)	
**Family tumor history**,** n(%)**					0.403
No	780(95.5)	608(95.7)	229(97.4)	1617(95.9)	
Yes	37(4.5)	27(4.3)	6(2.6)	70(4.1)	
**Hypertension**,** n(%)**					0.802
No	656(80.3)	506(79.7)	192(81.7)	1354(80.3)	
Yes	161(19.7)	129(20.3)	43(18.3)	333(19.7)	
**Diabetes**,** n(%)**					0.551
No	734(89.8)	568(89.4)	216(91.9)	1518(90.0)	
Yes	83(10.2)	67(10.6)	19(8.1)	169(10.0)	
**CEA**,** n(%)**					0.696
≤5	539(66.0)	414(65.2)	148(63.0)	1101(65.3)	
>5	278(34.0)	221(34.8)	87(37.0)	586(34.7)	
**T**,** n(%)**					<0.001
T1-2	96(11.8)	34(5.4)	8(3.4)	138(8.2)	
T3	627(76.7)	532(83.8)	164(69.8)	1323(78.4)	
T4	94(11.5)	69(10.9)	63(26.8)	226(13.4)	
**Differentiation**,** n(%)**					<0.001
Poor	149(18.2)	131(20.6)	65(27.7)	345(20.5)	
Median	507(62.1)	400(63.0)	151(64.3)	1058(62.7)	
Well	161(19.7)	104(16.4)	19(8.1)	284(16.8)	
**Histology**,** n(%)**					0.009
Adenocarcinoma	729(89.2)	552(86.9)	192(81.7)	1473(87.3)	
Other	88(10.8)	83(13.1)	43(18.3)	214(12.7)	
**Perinerual Invasion**,** n(%)**					<0.001
No	711(87.0)	580(91.3)	168(71.5)	1459(86.5)	
Yes	106(13.0)	55(8.7)	67(28.5)	228(13.5)	
**Vascular Invasion**,** n(%)**					<0.001
No	703(86.0)	557(87.7)	166(70.6)	1426(84.5)	
Yes	114(14.0)	78(12.3)	69(29.4)	261(15.5)	
**MMR status**,** n(%)**					<0.001
pMMR	665(81.4)	467(73.5)	213(90.6)	1345(79.7)	
dMMR	152(18.6)	168(26.5)	22(9.4)	342(20.3)	
**Chemotherapy**,** n(%)**					<0.001
No	475(58.1)	351(55.3)	60(25.5)	886(52.5)	
Adjuvant Chemotherapy	342(41.9)	284(44.7)	175(74.5)	801(47.5)	
**Number of Lymph Nodes Dissected**,** n**	28.39 ± 12.04	30.44 ± 13.22	28.05 ± 12.44	29.11 ± 12.59	0.003
**Positive Lymph Node**,** n**	3.73 ± 5.82	1.97 ± 1.48	3.82 ± 3.48	3.44 ± 4.47	0.001
**Positive Lymph Node Ratio(%)**	13.55 ± 17.53	7.74 ± 5.20	15.07 ± 13.92	13.07 ± 14.75	<0.001

“Other” in Histology refers to special types of adenocarcinoma mainly include mucinous adenocarcinoma, accounting for 191/214

(Abbreviations: BMI, Body Mass Index; CEA, Carcinoembryonic Antigen; MMR, mismatch repair; dMMR, deficient mismatch repair; pMMR, proficient mismatch repair)

### Clinical characteristics of the overstaged group

**Fig. 1:**
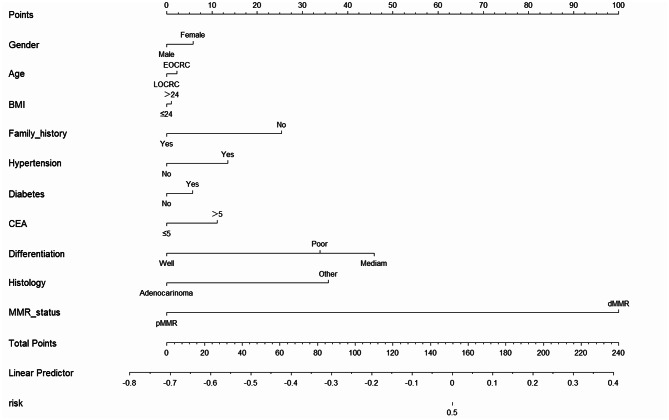
Nomogram predicting patients between the overstaged and consistent groups. (Abbreviations: EOCRC, Early-Onset Colorectal Cancer; LOCRC, Late-Onset Colorectal Cancer; BMI, Body Mass Index; CEA, Carcinoembryonic Antigen; MMR, mismatch repair; dMMR, deficient mismatch repair; pMMR, proficient mismatch repair; PNI, perineural invasion)

We compared the preoperative clinical data, including sex, age, body mass index (BMI), family tumor history, hypertension, diabetes, carcinoembryonic antigen (CEA) level, tumor differentiation, tumor histology, and MMR status, between the overstaged and consistent groups via univariate logistic regression analysis. We found that only the MMR status was significantly different between the two groups, indicating that patients with dMMR were more likely to be overstaged before surgery (odds ratio [OR], 1.551; 95% confidence interval [CI],1.210–1.989; *P* = 0.001, as shown in Table 2). Through the analysis of nomogram, we also found that the MMR status had the highest weight in estimating whether the preoperative cN stage was overstaged (Fig. 1).

**Table 2:** Univariate logistic regression analysis predicting patients between the overstaged and consistent groups

logistics		
	Univariate logistics model	
	Odds Ratio	*P*-value
	**(95% CI)**	
**Gender**		
Female		
Male	0.972(0.790–1.197)	0.791
**Age**		
≥ 50		
<50	1.027(0.830–1.272)	0.804
**BMI**		
<24		
≥ 24	0.998(0.795–1.252)	0.984
**Family tumor history**		
No		
Yes	0.936(0.564–1.555)	0.799
**Hypertension**		
No		
Yes	1.039(0.802–1.346)	0.774
**Diabetes**		
No		
Yes	1.043(0.742–1.466)	0.808
**CEA**		
≤ 5		
>5	1.035(0.832–1.287)	0.757
**Differentiation**		
Poor		
Median	0.897(0.686–1.174)	0.430
Well	0.735(0.523–1.033)	0.076
**Histology**		
Adenocarcinoma		
Other	1.246(0.905–1.715)	0.178
**MMR status**		
pMMR		
dMMR	1.574(1.227–2.019)	<0.001

(Abbreviations: BMI, Body Mass Index; CEA, Carcinoembryonic Antigen; MMR, mismatch repair; dMMR, deficient mismatch repair; pMMR, proficient mismatch repair)

### Prognostic analysis

**Fig. 2:**
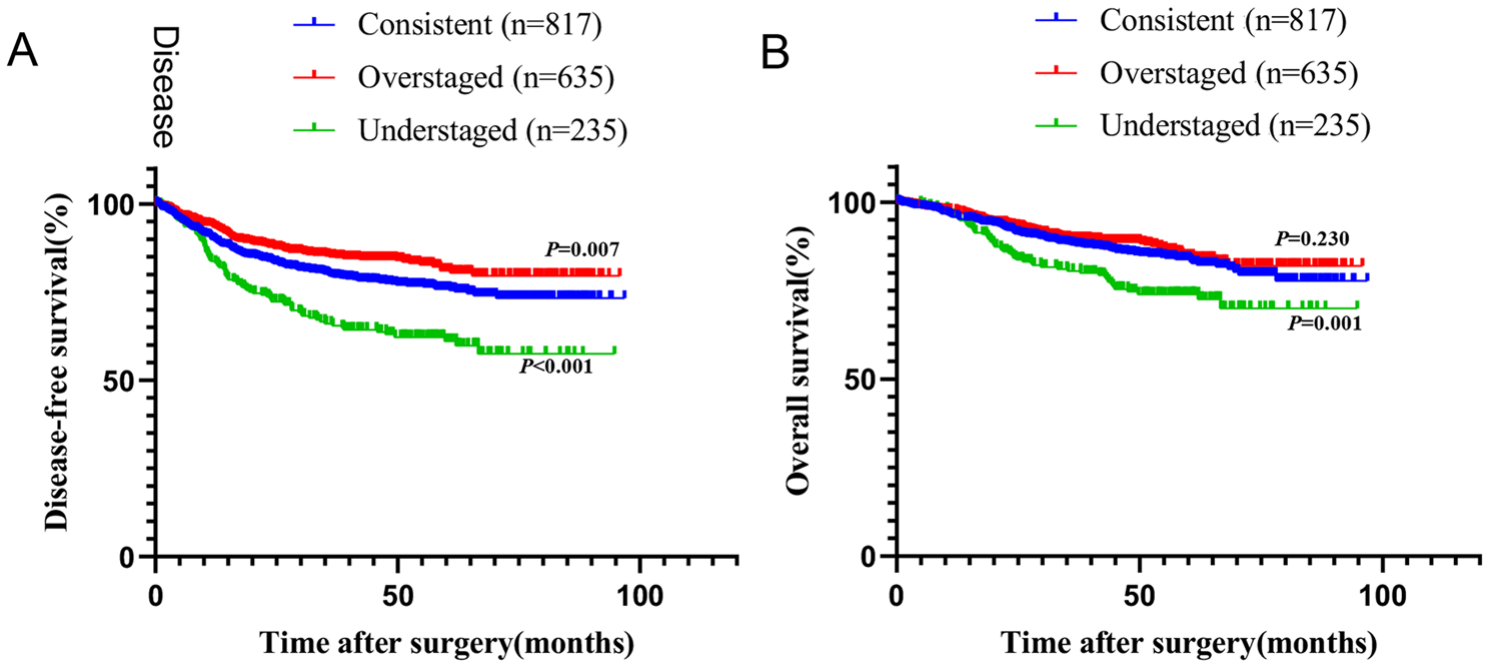
Prognostic analysis of patients with stage I-III right-sided colon cancer according to consistence between preoperative cN and postoperative pN. (**A**) Survival curves of DFS comparing consistent group, overstaged group and understaged group. (**B**) Survival curves of OS comparing consistent group, overstaged group and understaged group

The median follow-up time was 46.3 months (range 0.2–96.8 months). As shown in Fig. 2, the overstaged group had the best prognosis among the three groups in terms of disease-free survival (DFS) (*P* = 0.007), whereas the understaged group had the worst prognosis among the three groups in terms of overall survival (OS) (*P* = 0.001) and DFS (*P* < 0.001).

In the multivariate Cox regression analysis of DFS, CEA > 5 ng/mL, perineural invasion (PNI), dMMR, and adjuvant chemotherapy were found to be independent prognostic factors for DFS (Table 3). Moreover, dMMR status was a protective factor in the overstaged group (HR = 0.535, 95% CI: 0.307–0.932; *P* = 0.027). The results of the Cox proportional hazard model for OS are presented in Supplementary Table 2.

**Table 3:** Univariate and multivariate analysis of DFS in patients with stage I-III right-sided colon cancer

DFS				
	Univariate Cox model		Multivariate Cox model	
	Hazard Ratio	*P*-value	Hazard Ratio	*P*-value
	**(95% CI)**		**(95% CI)**	
**Gender**				
Female	1(ref)			
Male	1.017(0.689–1.501)	0.933		
**Age**				
≥50	1(ref)			
<50	0.885(0.597–1.313)	0.545		
**BMI**				
<24	1(ref)			
≥24	0.677(0.425–1.077)	0.099		
**Family tumor history**				
No	1(ref)			
Yes	0.196(0.027–1.404)	0.105		
**Hypertension**				
No	1(ref)			
Yes	1.190(0.747–1.894)	0.464		
**Diabetes**				
No	1(ref)			
Yes	0.614(0.285–1.322)	0.212		
**CEA**				
≤5	1(ref)		1(ref)	
>5	1.972(1.337–2.908)	0.001	1.599(1.068–2.392)	0.023
**T**				
T1-2	1(ref)		1(ref)	
T3	1.417(0.447–4.499)	0.554	1.323(0.411–4.257)	0.638
T4	4.594(1.390-15.182)	0.012	2.942(0.845–10.245)	0.090
**Differentiation**				
Poor	1(ref)			
Median	0.777(0.488–1.238)	0.289		
Well	0.732(0.391–1.373)	0.331		
**Histology**				
Adenocarcinoma	1(ref)			
Other	1.279(0.750–2.181)	0.367		
**Perinerual Invasion**				
No	1(ref)		1(ref)	
Yes	4.051(2.560–6.412)	<0.001	2.572(1.516–4.365)	<0.001
**Vascular Invasion**				
No	1(ref)		1(ref)	
Yes	2.284(1.422–3.669)	0.001	1.479(0.877–2.496)	0.142
**MMR status**				
pMMR	1(ref)		1(ref)	
dMMR	0.455(0.263–0.787)	0.005	0.535(0.307–0.932)	0.027
**Chemotherapy**				
No	1(ref)		1(ref)	
Adjuvant Chemotherapy	0.659(0.440–0.987)	0.043	0.538(0.358–0.808)	0.003

(Abbreviations: DFS, disease-free survival; BMI, Body Mass Index; CEA, Carcinoembryonic Antigen; MMR, mismatch repair; dMMR, deficient mismatch repair; pMMR, proficient mismatch repair)

## Discussion

This retrospective cohort study investigated the accuracy of preoperative N staging with CT in right-sided colon cancer patients. In this study, we found that the specificity of N staging in patients with right-sided colon cancer was 51.8%, which was consistent with previous studies [3, 5]. Among the 870 cases with an incorrect N stage, the N stage of 635 cases (73.0%) was overestimated, similar to the findings of a previous study [15]. As far as we known, our work included the largest cohort of right-sided colon cancer integrating MMR status and preoperative CT nodal staging. We believed that our study could provide more evidence in the field. Moreover, we emphasized the prognostic value of understaging and overstaging group in right-sided colon cancer, especially understaging patients had worst outcomes. The clinical effects of N overstaging include radical surgery for lymph node dissection and excessive chemotherapy. Recent research has shown that regional lymph nodes play an important role in antitumor immunity, requiring care to be taken regarding excessive nonmetastatic lymph node dissection in dMMR CRCs, which may have a positive effect on the long-term prognosis of dMMR CRC patients [16]. With the use of neoadjuvant chemotherapy [4, 17] and immunotherapy [18] for colon cancer, accurate N staging will have a greater impact on treatment decisions.

The specificity of N staging in patients with right-sided colon cancer was 51.8%, whereas that in patients with dMMR was 44.0%. The N stage tends to be overstaged in patients, especially in dMMR patients. As shown in a previous study, significantly higher levels of cytotoxic T cells were observed in the tumor and peritumoral regions of microsatellite instable (MSI) tumors than in microsatellite stable (MSS) tumors [19]. This inflammatory pattern may explain the greater number of nonmetastatic regional lymph nodes and N-stage overstaging in patients with dMMR. A recent study revealed significant differences in the radiological appearance of lymph nodes between dMMR and pMMR patients [14]. We also analyzed the prognostic influence of overstaging and understaging and found that overstaging was associated with best prognosis while understaging tended to have worst prognosis. Imaging assessment might overstage lymph nodes in order to avoid insufficient treatment in clinical practice, which caused poor prognosis. This prompted us to assess the CT-based N stage considering the patient’s MMR status. Patients with dMMR may require a more effective method for assessing lymph node metastasis.

In our study, overstaged patients showed better DFS, while understaged patients showed worst DFS and OS, which may indicate that we should strengthen treatment and follow up more frequently for those patients with understaged CT N stage in clinical practise. Furthermore, CEA level > 5 ng/mL, PNI, dMMR, and adjuvant chemotherapy were found to be independent prognostic factors for OS. Among these factors, the dMMR is an essential protective factor. This may be because dMMR is a protective factor against stage II colon cancer [20], which has been clearly stated in multiple guidelines [21, 22]. For overstaged patients with CEA level > 5 ng/mL or PNI, we recommend close follow-up to monitor tumor recurrence.

Our study had several limitations that warrant further investigation. First, its retrospective design is a significant source of bias. Another potential source of bias is pathology reports from single institutions without a complete review, and the study did not require specific expertise in the review of lymph nodes. Third, this study only focused on right-sided colon cancer, which should not be extrapolated to non-right-sided colon cancer, due to the difference of MMR/MSI prevalence and nodal patterns.

## Conclusion

In conclusion, the N stage via CT scans tends to be overstaged in patients with right-sided colon cancer, especially in patients with dMMR. The assessment of CT-based lymph node staging should take tumour MMR status into account, as dMMR right-sided colon cancers are more prone to CT-based nodal overstaging. This awareness may help to contextualise borderline imaging findings and to avoid overtreatment in selected patients, while recognising that clinically suspicious lymph nodes cannot be disregarded solely based on dMMR status. For prognosis of patients with right-sided colon cancer, dMMR and adjuvant chemotherapy were independent protective factors, whereas CEA > 5 ng/mL and PNI were risk factors for overstaged patients.

## Supplementary Information

Below is the link to the electronic supplementary material.

Supplementary Material 1: Supplementary Fig. 1 Flow chart of enrolled patients.

Supplementary Material 2: Supplementary Table 1 The accuracy of CT-based cN stage.

Supplementary Material 3: Supplementary Table 2 Univariate and multivariate analysis of OS in patients with stage I-III right-sided colon cancer.

## Data Availability

All relevant data are within the paper and its Supporting Information files.

## Abbreviations

CT: Computed tomography
OR: Odds ratio
CI: Confidence interval
CRC: Colorectal cancer
MMR: Mismatch repair
dMMR: Deficient mismatch repair
pMMR: Proficient mismatch repair
DFS: Disease-free survival
OS: Overall survival
PNI: Perineural invasion
