# Supplementary material for: Impact of MMR status on preoperative CT-based lymph node overstaging in right-sided colon cancer: a retrospective analysis
Source: Cancer Imaging. 2026 Feb 5;26:22. doi: 10.1186/s40644-026-00992-3 (PMC12874889; doi:10.1186/s40644-026-00992-3)
Supplement: Supplementary file 1 — Supplementary Material 1: Supplementary Fig. 1 Flow chart of enrolled patients. [file 40644_2026_992_MOESM1_ESM.docx]

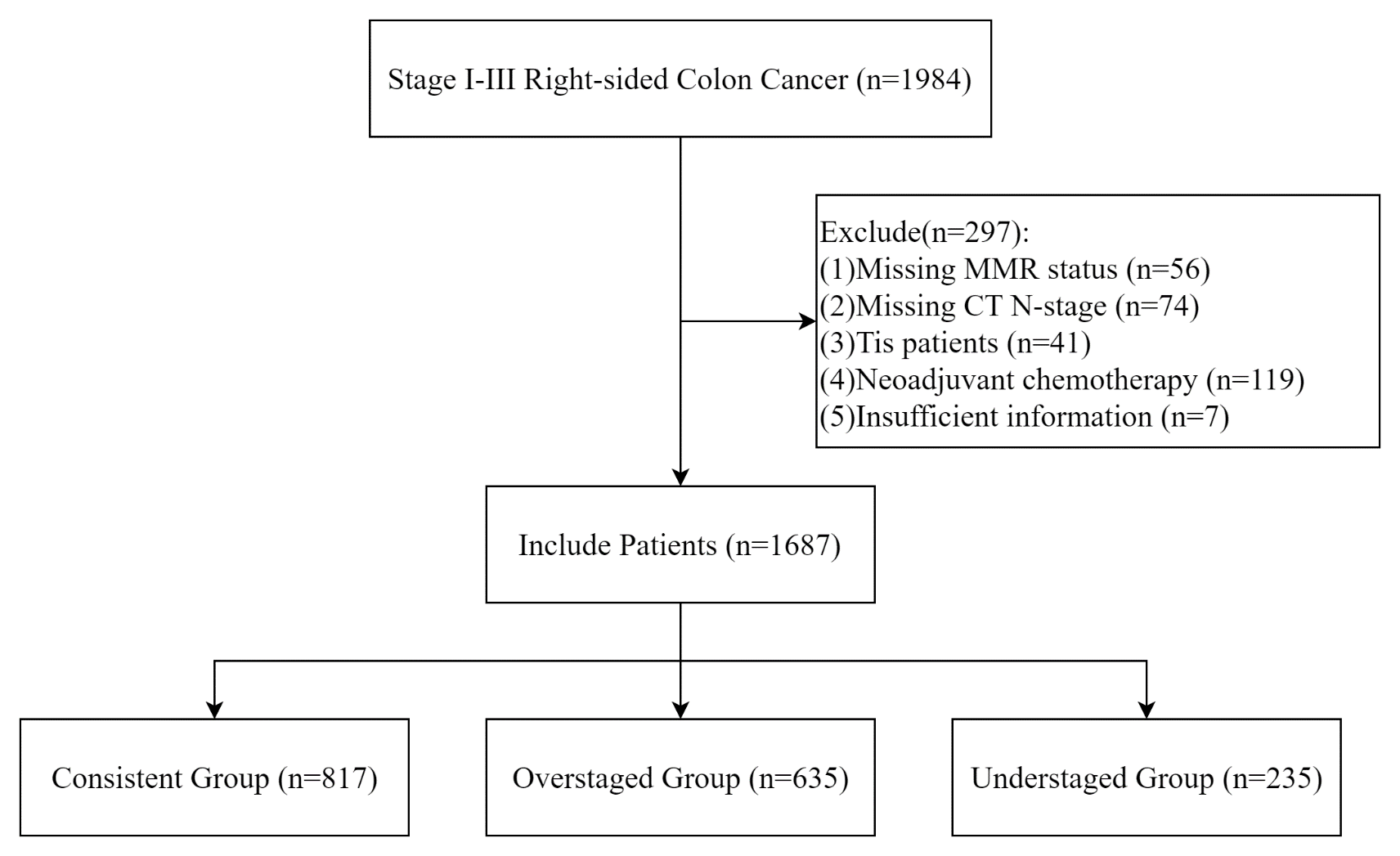


Supplementary Figure 1 Flow chart of enrolled patients. (Abbreviations: MMR, mismatch repair; Tis, Tumor in situ)
