# Supplementary material for: Impact of MMR status on preoperative CT-based lymph node overstaging in right-sided colon cancer: a retrospective analysis
Source: Cancer Imaging. 2026 Feb 5;26:22. doi: 10.1186/s40644-026-00992-3 (PMC12874889; doi:10.1186/s40644-026-00992-3)
Supplement: Supplementary file 2 — Supplementary Material 2: Supplementary Table 1 The accuracy of CT-based cN stage. [file 40644_2026_992_MOESM2_ESM.docx]

Supplementary Table 1 The accuracy of CT-based cN stage.

|  |  | **p*N*positive** | **p*N*negative** | **Total** |  |
| --- | --- | --- | --- | --- | --- |
| **Total** | **CT*N*positive** | 417 | 523 | 940 | Sensitivity:69.3%(n=417/601)  Specificity:51.8%(n=563/1086)  PPV:44.3%(n=417/940)  NPV:75.3%(n=563/747) |
|  | **CT*N*negative** | 184 | 563 | 747 |  |
|  | **Total** | 601 | 1086 | 1687 |  |
| **pMMR** | **CT*N*positive** | 362 | 372 | 734 | Sensitivity:68.4%(n=362/529)  Specificity:54.4%(n=444/816)  PPV:49.3%(n=362/734)  NPV:72.6%(n=444/611) |
|  | **CT*N*negative** | 167 | 444 | 611 |  |
|  | **Total** | 529 | 816 | 1345 |  |
| **dMMR** | **CT*N*positive** | 55 | 151 | 206 | Sensitivity:76.3%(n=55/72)  Specificity:44.0%(n=119/270)  PPV:26.6%(n=55/206)  NPV:87.5%(n=119/136) |
|  | **CT*N*negative** | 17 | 119 | 136 |  |
|  | **Total** | 72 | 270 | 342 |  |

CTNnegative=CTN0, CTNpositive=CTN1+CTN2, pNnegative=pN0, pNpositive=pN1+pN2, PPV: Positive predictive value, NPV: Negative predictive value.
