# Supplementary material for: Impact of MMR status on preoperative CT-based lymph node overstaging in right-sided colon cancer: a retrospective analysis
Source: Cancer Imaging. 2026 Feb 5;26:22. doi: 10.1186/s40644-026-00992-3 (PMC12874889; doi:10.1186/s40644-026-00992-3)
Supplement: Supplementary file 3 — Supplementary Material 3: Supplementary Table 2 Univariate and multivariate analysis of OS in patients with stage I-III right-sided colon cancer. [file 40644_2026_992_MOESM3_ESM.docx]

Supplementary Table 2 Univariate and multivariate analysis of OS in patients with stage I-III right-sided colon cancer.

| **OS** | | | | | |
| --- | --- | --- | --- | --- | --- |
|  | **Univariate Cox model** | |  | **Multivariate Cox model** | |
|  | **Hazard Ratio** | **P-value** |  | **Hazard Ratio** | **P-value** |
|  | **(95% CI)** |  |  | **(95% CI)** |  |
| **Gender** |  |  |  |  |  |
| Female | 1(ref) |  |  |  |  |
| Male | 1.030(0.656-1.618) | 0.898 |  |  |  |
| **Age** |  |  |  |  |  |
| ≥50 | 1(ref) |  |  |  |  |
| ＜50 | 0.822(0.522-1.293) | 0.396 |  |  |  |
| **BMI** |  |  |  |  |  |
| ＜24 | 1(ref) |  |  |  |  |
| ≥24 | 0.637(0.367-1.106) | 0.109 |  |  |  |
| **Family tumor history** |  |  |  |  |  |
| No | 1(ref) |  |  |  |  |
| Yes | 0.258(0.036-1.855) | 0.178 |  |  |  |
| **Hypertension** |  |  |  |  |  |
| No | 1(ref) |  |  |  |  |
| Yes | 1.415(0.841-2.382) | 0.191 |  |  |  |
| **Diabetes** |  |  |  |  |  |
| No | 1(ref) |  |  |  |  |
| Yes | 0.746(0.324-1.718) | 0.491 |  |  |  |
| **CEA** |  |  |  |  |  |
| ≤5 | 1(ref) |  |  | 1(ref) |  |
| ＞5 | 2.157(1.374-3.386) | 0.001 |  | 1.907(1.202-3.027) | 0.006 |
| **T** |  |  |  |  |  |
| T1-2 | 1(ref) |  |  |  |  |
| T3 | 0.927(0.289-2.969) | 0.898 |  |  |  |
| T4 | 3.186(0.946-10.727) | 0.061 |  |  |  |
| **Differentiation** |  |  |  |  |  |
| Poor | 1(ref) |  |  |  |  |
| Median | 0.938(0.531-1.656) | 0.824 |  |  |  |
| Well | 1.003(0.489-2.058) | 0.993 |  |  |  |
| **Histology** |  |  |  |  |  |
| Adenocarinoma | 1(ref) |  |  |  |  |
| Other | 1.223(0.660-2.267) | 0.522 |  |  |  |
| **Perinerual Invasion** |  |  |  |  |  |
| No | 1(ref) |  |  | 1(ref) |  |
| Yes | 4.349(2.581-7.328) | ＜0.001 |  | 3.812(2.149-6.760) | ＜0.001 |
| **Vascular Invasion** |  |  |  |  |  |
| No | 1(ref) |  |  | 1(ref) |  |
| Yes | 2.366(1.359-4.120) | 0.002 |  | 1.782(0.978-3.246) | 0.059 |
| **MMR status** |  |  |  |  |  |
| pMMR | 1(ref) |  |  | 1(ref) |  |
| dMMR | 0.500(0.270-0.926) | 0.027 |  | 0.592(0.316-1.108) | 0.101 |
| **Chemotherapy** |  |  |  |  |  |
| No | 1(ref) |  |  | 1(ref) |  |
| Adjuvant Chemotherapy | 0.496(0.304-0.810) | 0.005 |  | 0.391(0.238-0.642) | ＜0.001 |

(Abbreviations: OS, overall survival; BMI, Body Mass Index; CEA, Carcinoembryonic Antigen; MMR, mismatch repair; dMMR, deficient mismatch repair; pMMR, proficient mismatch repair)
